# IL-37 alleviates liver granuloma caused by *Schistosoma japonicum* infection by inducing alternative macrophage activation

**DOI:** 10.1186/s13071-022-05420-6

**Published:** 2022-08-24

**Authors:** Cuiping Ren, Fengchun Liu, Chen Xing, Ruyu Zhao, Xiaoxue Tang, Miao Liu, Wenda Gao, Jijia Shen

**Affiliations:** 1 Department of Microbiology and Parasitology, Anhui Provincial Laboratory of Pathogen Biology; Anhui Provincial Laboratory of Zoonoses; Laboratory of Tropical and Parasitic Diseases Control; School of Basic Medical Sciences, Anhui Medical University, Hefei, 230032 China; 2 Anhui Provincial Center for Clinical Laboratories, The First Affiliated Hospital of University of Science and Technology of China, Hefei, 230032 China; 3 Antagen Institute for Biomedical Research, Boston, MA 02118 USA

**Keywords:** *Schistosoma japonicum*, IL-37, Liver granuloma, Macrophages, AMPK

## Abstract

**Background:**

Hepatic macrophages regulate liver granuloma formation and fibrosis caused by infection with* Schistosoma japonicum*, with the manner of regulation dependent on macrophage activation state. Interleukin (IL)-37 may have immunomodulatory effects on macrophages. However, whether IL-37 can affect liver granuloma formation and fibrosis by affecting the polarization of macrophages in *S. japonicum* infection remains unclear. The aim of this study was to investigate IL-37-affected macrophage polarization in liver granuloma formation and fibrosis in *S. japonicum* infection.

**Methods:**

An enzyme-linked immunosorbent assay (ELISA) was used to detect the expression of IL-37 in the serum of patients with acute *S. japonicum* infection and in the serum of healthy people. Recombinant IL-37 (rIL-37), CPP-IgG_2_Fc-IL-37 and no CPP-IgG_2_Fc-IL-37 proteins were injected into *S. japonicum*-infected mice every 3 days for a total of 6 times from day 24 post infection onwards. Subsequently, ELISA, quantitative reverse transcription-PCR, fluorescence-activated cell sorting and western blot were used to analyze whether IL-37 inhibits the formation of liver granulomas and the development of liver fibrosis by regulating the phenotypic transition of macrophages. Finally, the three IL-37 proteins and SIS3, a Smad3 inhibitor, were co-cultured in mouse peritoneal macrophages to explore the mechanism underlying the promotion of the polarization of M0 macrophages to the M2 phenotype by IL-37.

**Results:**

Serum IL-37 levels were upregulated in schistosomiasis patients, and this increased level of IL-37 protein apparently alleviated the liver granuloma of mice in infection models. It also could induce liver and peritoneal macrophages to polarize to the M2 phenotype in *S. japonicum*-infected mice. The *S. japonicum*-infected mice injected with CPP-IgG_2_Fc-IL-37 group exhibited the most obvious improvement in inflammatory reaction against the liver granuloma. The number and ratio of M2 macrophages in the liver and peritoneal cavity were significantly higher in the three IL-37 protein groups, especially in the CPP-IgG_2_Fc-IL-37 group, compared to the controls. Similar results were also found regarding liver function damage. IL-37 induced macrophage M2 polarization by promoting AMP-activated protein kinase (AMPK) phosphorylation in vitro. Among all groups, the activation of AMPK was most significant in the CPP-IgG_2_Fc-IL-37 group, and it was found that SMAD3 could enhance the anti-inflammatory function of IL-37.

**Conclusions:**

The results show that IL-37 was able to promote the polarization of macrophages to the M2 phenotype, thereby inhibiting the development of schistosomiasis. In comparison to the rIL-37 protein, the CPP-IgG_2_Fc-IL-37 protein has the advantages of being effective in small doses and having fewer side effects and a better efficacy.

**Graphical Abstract:**

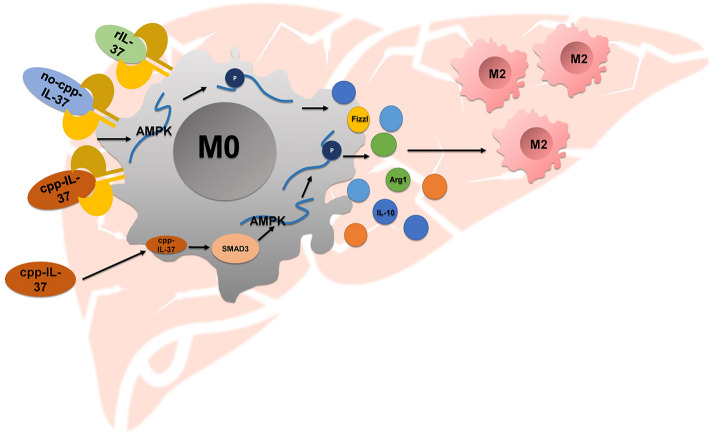

**Supplementary Information:**

The online version contains supplementary material available at 10.1186/s13071-022-05420-6.

## Background

The most serious clinical-pathological features of schistosomiasis include egg granuloma formation and hepatic fibrosis formed by egg deposition in the liver. As the deposited ova exert sustained antigenic stimulation, inflammatory and immune cells are sequentially recruited to the infection sites, leading to the granuloma formation and eventually chronic fibrosis in some infected individuals [1]. Generally, granulomas consist of macrophage clusters, including epithelioid and multinucleate giant cells [2]. Macrophages are not only the main cellular component of the granulomatous response to the eggs but they can also present antigens to initiate and regulate the adaptive immune response. They play a key role in the formation and development of egg granulomas and hepatic fibrosis [3, 4]. There are two main subtypes of macrophages: M1 macrophages (classically activated macrophages) and M2 macrophages (alternatively activated macrophages) [3]. M1 macrophages are antigen-presenting cells which activate the adaptive immune response by secreting pro-inflammatory cytokines and chemokines, such as inducible nitric oxide synthase (iNOS), C-X-C motif chemokine 9 (CXCL9) and tumor necrosis factor alpha (TNF-α). M2 macrophages can be alternatively activated following induction by granulocyte-macrophage colony-stimulating factor (GM-CSF), interleukin (IL)-4, tumor growth factor beta (TGF-β), among others. Compared to M1 macrophages, M2 macrophages exhibit a lower antigen presentation ability, but they can downregulate the adaptive immune response through the secretion of anti-inflammatory cytokines, such as mannose receptor, arginase-1, IL-10, among others [5–7]. Macrophage polarization is a dynamically reversible process during schistosome infection, with the phenotype of macrophages changing as the inflammatory response progresses [4]. Hence, it is necessary to understand the causes of macrophage polarization during schistosome infection in depth.

IL-37, a new member of the IL-1 family, acts as a novel anti-inflammatory cytokine in various inflammatory and immune diseases [8–10]. It exhibits anti-inflammatory effects by inhibiting the production of inflammatory cytokines and promoting the release of anti-inflammatory cytokines [11]. Mature IL-37 can be secreted extracellularly and it binds to the two receptor subunits, SIGIRR and IL-18Rα, for negative immune regulation through the receptor pathway. It can also enter the nucleus and suppress immune response by interacting with the SMAD3 protein through nuclear transcription factor pathways [11–13]. Several innate immune cells can secrete IL-37 including, in particular, macrophages [14]. Luo et al. found that IL-37 can boost the polarization of macrophages from the pro-inflammatory M1 to the anti-inflammatory M2 phenotype, which is a promising treatment strategy for the treatment of temporomandibular joint inflammation [15]. However, to our knowledge, no research has linked IL-37 to the function of macrophages in egg granuloma formation and hepatic fibrosis during schistosome infection. Further, the mechanisms underlying the effect of IL-37 on macrophage activation are worth exploring. To facilitate such research, we designed, synthesized, and purified the protein CPP-IgG_2_Fc-IL-37 and protein no CPP-IgG_2_Fc-IL-37 expressed by eukaryotic cells. The CPP-IgG_2_Fc mut-IL-37 and no CPP-IgG_2_Fc mut-IL-37 are based on stably transfected adherent Chinese hamster ovary (CHO) cells, and the IL-37 protein is a human IL-37 protein. The CPP (cell-penetrating peptide) component possibly aids in the transport of proteins inside the cells. The IgG_2_Fc mutant section could avoid effector functions such as antibody-dependent cell-mediated cytotoxicity and complement-dependent cytotoxicity. We designed the caspase-1 cleavage site with the WEHD sequence using IL-37-CPP and IgG_2_Fc, which could help IL-37 enter the nucleus.

The aim of this study was to elucidate how IL-37 promotes the polarization of macrophages to the M2 phenotype in the early stage of schistosome infection. We also aimed to verify whether IL-37 can inhibit the inflammatory response of the host egg granuloma by inducing the phenotypic transition of macrophages. The results of this study may aid in identifying a potential therapeutic target for the prevention and treatment of schistosomiasis.

## Methods

### Serum sample data

Sera of 14 patients with acute schistosomiasis were provided by the Anhui Institute of Parasitic Diseases. The patients all came from Puqiao Village, Yijiang Town, Nanling, Wuhu, Anhui Province (China), which is an endemic area of schistosomiasis. We also collected the sera of 14 healthy people (controls), who were originally from non-endemic areas of schistosomiasis, at the Anhui Medical University.

A confirmed case of acute *S. japonicum* infection was based on the following criteria formulated by the Ministry of Public Health in China [16]: (i) positive stool examination result for *S. japonicum* eggs using the Kato-Katz method; (ii) positive serology for schistosomiasis; (iii) recent history of exposure to water; (iv) fever and other relevant symptoms; and (v) peripheral blood eosinophilia comprising ≥ 15% of the total leukocyte count.

### Preparation of infection models of *S. japonicum*

Six-week-old female C57BL/6 mice were purchased from the Experimental Animal Center of Anhui Province in Hefei, China. Healthy mice were randomly divided into five groups, with five mice in each group. Each mouse was infected percutaneously with 20 ± 1 *S. japonicum* cercariae. Freshwater snails (*Oncomelania hupensis*) harboring *S. japonicum* cercariae were purchased from the Hunan Institute of Parasitic Diseases in Yueyang, China. Three different IL-37 proteins (i.e. rIL-37 [recombinant IL-37], CPP-IgG2Fc-IL-37 and no CPP-IgG2Fc-IL-37 proteins) were injected into the tail vein every 3 days for a total of 6 times from day 24 post infection onwards.

### Enzyme-linked immunosorbent assay

The sera were tested by double-antibody sandwich enzyme-linked immunosorbent assay (ELISA) for the indicated cytokines. A human IL-37 kit was purchased from Beijing 4A Biotech Co., Ltd (Beijing, China).

### Detection of alanine aminotransferase and aspartate aminotransferase

Blood was collected from the mouse’s eyeballs, placed at 37 °C for 2 h and centrifuged at 1000 *g* for 5 min. The upper layer (serum) was removed and stored at − 80 °C. The alanine aminotransferase (ALT) and aspartate aminotransferase (AST) levels in the serum were detected using ALT and AST assay Kits, respectively, from the Nanjing Jiancheng Biotechnology Co., Ltd (Nanjing, China).

### Egg count

Approximately 0.1 g of the left lobe of mouse liver tissue was taken for further analysis. Eggs in the liver were counted after digestion for 3 h at 37 °C in 5% KOH. The eggs present in 0.1 ml of the digested tissues were quantified by smear examination under the microscope (×20; Olympus, Tokyo, Japan), which was repeated 5 times. A morphological analysis system (JEDA-801D; Jiangsu JEDA Science-Technology Development Co., Ltd.) was used to estimate the size of 120 eggs from each group.

### Hematoxylin and eosin staining

Liver samples were fixed with 4% (w/v) paraformaldehyde, embedded in paraffin, cut into 4-μm-thick sections and stained with hematoxylin and eosin (H&E) for morphological investigations.

### Separation and stimulation of the liver egg granuloma cells

Granulomas from the livers of *S. japonicum*-infected mice and from the IL-37 treatment group were included in this study. Liver granuloma cells were separated as previously described [17]. Briefly, liver tissues were cut into small piece and homogenized in a glass homogenizer. The granulomas were collected after precipitation and washing in RPIM-1640 medium (Gibco, Thermo Fisher Scientific, Waltham, MA, USA), then digested in 10% fetal bovine serum (FBS; Gibco) containing type IV collagenase (Sigma-Aldrich, St. Louis, MO, USA) in a shaker at 37 °C and 210 rpm for 15 min. The granulomas were then digested again for 15 min after filtering through 200-gauge steel screens. The digestion reaction was terminated by adding 5 ml RPMI-1640 medium to the solution and placing it on ice for 5 min. The filtrate was centrifuged at 2000*g* for 15 min after filtering through 200-gauge steel screens. Finally, the pellet was resuspended in RPMI-1640 medium containing 10% FBS.

The liver egg granuloma cells were separated as described above for the granulomas and inoculated into a 24-well plate at a density of 1 × 10^6^/ml soluble egg antigen (SEA, 50 μg/ml) was added to each well with fresh medium. The cells were incubated for 4 h at 37 °C, followed by the addition of 25 ng/ml phorbol12-myristate13-acetate (PMA, Sigma-Aldrich), 1 μg/ml ionomycin (Sigma-Aldrich) and 10 μg/ml brefeldin A (BFA, Sigma-Aldrich) to each well. The cells were collected after 48 h of culture.

### Peritoneal macrophage separation and culture

The peritoneal exudate cells were collected by lavage of the peritoneal cavity with 5 ml Dulbecco’s Modified Eagle Medium (DMEM) medium, followed by centrifugation. The cells were resuspended in ACK lysis buffer for erythrocyte lysis and centrifuged at 300 *g* for 10 min. To remove non-adherent cells after incubating for 2 h, the cells were resuspended in DMEM medium containing 10% FBS and 1% penicillin–streptomycin and inoculated into a 6-well plate at a density of 5 × 10^6^/ml. The peritoneal macrophages were subsequently re-seeded into a 24-well plate and stimulated with different concentrations of CPP-IgG_2_Fc-IL-37, no CPP-IgG_2_Fc-IL-37, or recombinant IL-37 (rIL-37) protein, respectively.

### Flow cytometry

Single-cell suspensions were washed in phosphate-buffered saline (PBS) with 1% FBS and adjusted to 1 × 10^6^ cells per 100 μl PBS with 1% FBS. For the purity analysis of macrophages, cells were incubated with peridinin chlorophyll protein-Cy5.5 conjugated antibody against mouse F4/80 (BioLegend, San Diego, CA, USA) and FITC-conjugated antibody against mouse CD11b (Biolegend). For M1 and M2 surface marker analysis, cells were incubated with PE-conjugated antibodies against mouse iNOS (eBioscience, San Diego, CA, USA) and APC-conjugated antibodies against mouse CD206 (Biolegend). All antibodies were used at 5 μg/ml, and the cells were incubated with the antibodies for 30 min at 4 °C, followed by washing with PBS. The cells were fixed with 3% paraformaldehyde. Finally, all cells were examined using a flow cytometer (BD Biosciences, San Jose, CA, USA), and the results were analyzed using FlowJo7.6 software (Tree Star Inc., Ashland, OR, USA).

### RNA extraction and quantitative PCR

Total RNA was extracted from liver tissues and macrophages utilizing TRIzol reagent and reverse transcribed into complementary DNA (cDNA) with TB Green™ Premix ExTaq™ II (Takara, Dalian, China). Quantitative PCR was used to examine the expression levels of *iNOS*, *Il1b*, *Tnf, Fizzl, Arg* and *Il10* using the PrimeScript™ RT Reagent Kit with the StepOnePlus™ Real Time PCR System (Applied Biosystems, Foster City, CA, USA). The glyceraldehyde 3-phosphate dehydrogenase gene (*Gapdh*) was used as a normalized control. The primer pairs used in this experiment are as follows: (i) *iNOS*: forward, 5ʹ-TGCCCCTTCAATGGTTGGTA-3ʹ; reverse, 5ʹ-ACTGGAGGGACCAGCCAAAT-3ʹ; (ii) *Il1b*: forward, 5ʹ-CTGAACTCAACTGTGAAATGC-3ʹ; reverse, 5ʹ-TGATGTGCTGCTGCGAGA-3ʹ; (iii) *Tnf*: forward, 5ʹ-ACTGGCAGAAGAGGCACTC-3ʹ; reverse, 5ʹ-CTGGCACCACTAGTTGGTTG-3ʹ; (iv) *Fizzl*: forward, 5ʹ-CCCTCCACTGTAACGAAGACTC-3ʹ; reverse, 5ʹ-CACACCCAGTAGCAGTCATCC-3ʹ; (v) *Arg*: forward, 5ʹ-CAGAAGAATGGAAGAGTCAG-3ʹ; reverse, 5ʹ-CAGATATGCAGGGAGTCACC-3ʹ; (vi) *Il-10*: forward, 5ʹ-TGAAGACCCTCAGGATGCGG-3ʹ; reverse, 5ʹ-AGAGCTCTGTCTAGGTCCTGG-3ʹ.

### Western blotting

Total protein was extracted from peritoneal macrophages using RIPA lysis buffer (Beyotime, Shanghai, China) with phenylmethanesulfonyl (PMSF; Beyotime) and cocktail (Sigma-Aldrich). The total protein in each sample was separated by sodium dodecyl sulfate-polyacrylamide gel electrophoresis and then transferred to nitrocellulose filter membranes (HATF00010; MilliporeSigma, Burlington, MA, USA). The membranes were incubated with primary antibodies at 4 °C after blocking with 5% skim milk. The primary antibodies were rabbit anti-AMP-activated protein kinase (AMPK) alpha1 (phospho T183) + AMPK alpha2 (phospho T172) (Abcam, Cambridge, UK), rabbit anti-AMPK (Abcam) and mouse anti-β-actin (Abcam). On the following day, the membranes were incubated with the horseradish peroxidase-conjugated goat anti-rabbit immunoglobulin G (IgG) or goat anti-mouse IgG (Zsbio, Beijing, China) at 25 °C for 1 h. The images were acquired using a protein imaging system (Proteinsimple, lourCherm FC3, China).

### Statistical analysis

All data were analyzed using the SPSS 22.0 software package (SPSS IBM, Armonk, NY, USA). Results are presented as the mean ± standard deviation for at least three independent experiments for each group. Each result was obtained from triplicate samples and following the same protocol. The differences between groups were assessed by Student’s t-test or one-way analysis of variance. *P* values < 0.05 were considered to be statistically significant.

## Results

### Serum IL-37 level is upregulated in patients with schistosomiasis

**Fig. 1:**
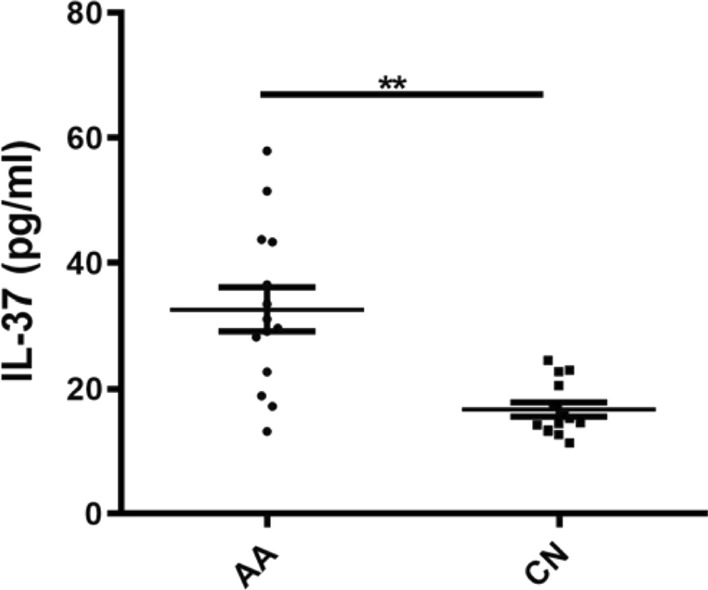
Expression levels of IL-37 in serum obtained from persons with AA or from CN, as detected by enzyme-linked immunosorbent assay. Data represent the mean ± SD from three independent experiments. Asterisks indicate significant difference at **P < 0.01. Abbreviations: AA, Patients with acute schistosomiasis infection; CN, healthy people from non-endemic areas (controls); IL, interleukin; SD, standard deviation

To investigate the potential role of IL-37 in patients with schistosomiasis, we recruited 14 acute patients with schistosomiasis and 14 age- and gender-matched healthy controls into this study. In comparison with the healthy control group, the level of serum IL-37 expression was significantly increased in patients with schistosomiasis (*F*_(1,13)_ = 15.81, *P* = 0.002; Fig. 1). This result suggests that IL-37, as an anti-inflammatory cytokine, is associated with the pathogenesis and progression of schistosomiasis.

### IL-37 administration alleviates egg-induced liver granuloma inflammation

**Fig. 2:**
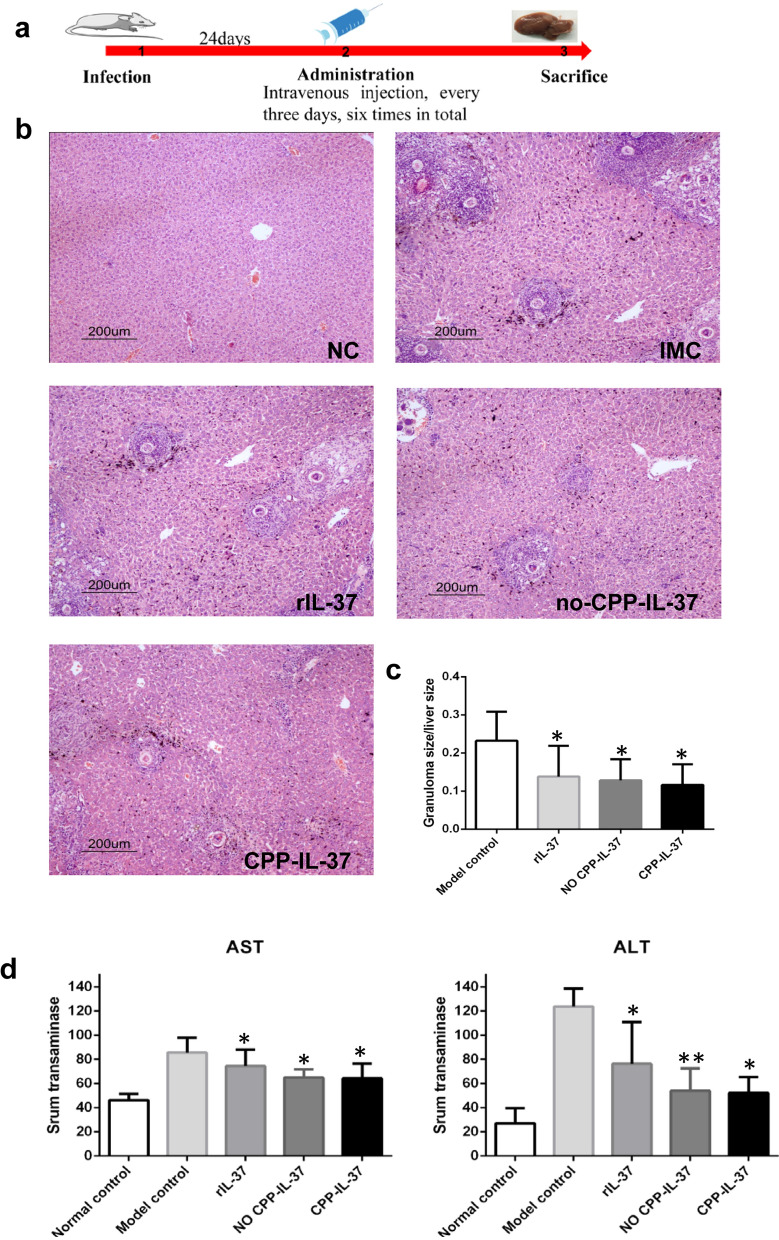
Administration of IL-37 alleviates egg-induced liver granuloma inflammation. **a** Dosing regimen used in the mouse model of liver egg granuloma formation. **b** Histological (H&E staining) images of liver egg granuloma formation from mice infected with *Schistosoma japonicum* or infected mice treated with IL-37. **c** Statistics chart of hepatic egg granuloma size/total area of mice livers is shown, 10 mice per each group. Asterisk indicates significant difference at *P < 0.05 versus the model control. **d** serum ALT/AST levels in mice infected with *S. japonicum* or IL-37 treatment group. Data represent the mean ± SD from 3 independent experiments, 5 mice per group. Asterisks indicate significant difference at *P < 0.05, **P < 0.01 versus the model control. Abbreviations: NC, Normal mice group; IMC, infection model control groups; rIL-37, infected mice livers after treatment with recombinant IL-37; CPP, cell-penetrating peptide; no CPP-IL-37, the infected mice livers after no CPP-IL-37 treatment; CPP-IL-37, infected mice livers after CPP-IL-37 treatment; ALT/AST, alanine aminotransferase and aspartate aminotransferase. Scale bars: 200 μm. Magnification: ×100

To investigate the effect of IL-37 on egg-induced hepatic granuloma formation in the early stage of schistosomiasis, C57BL/6 mice were used to prepare a *S. japonicum* infection model. Briefly, the mice were injected with CPP-IgG_2_Fc-IL-37, no CPP-IgG_2_Fc-IL-37, or rIL-37 through the tail vein before being sacrificed. The infection process is shown in Fig. 2a. There was no obvious change in the parasite and egg burden in the different groups of mice (Additional file 1: Table S1). However, IL-37 administration considerably improved the appearance of the liver, with H&E staining revealing that the infiltration of inflammatory cells and the hepatic egg-induced granuloma areas were significantly deceased in the three mice groups administered IL-37 (*P < 0.05; Fig. 2b, c). Although the group receiving CPP-IgG_2_Fc-IL-37 showed the greatest reduction in granuloma area, there was no statistical significance between the three IL-37 groups.

ALT and AST are sensitive indicators of liver function damage. We found that the serum levels of AST and ALT were lower in the group administered IL-37 than in the infected control group (**P < 0.01, *P < 0.05; Fig. 2d). The liver function damage in the no CPP-IgG_2_Fc-IL-37 or CPP-IgG_2_Fc-IL-37 group was not as severe as in the rIL-37 group. These results suggest that the CPP-IgG_2_Fc-IL-37 and no CPP-IgG_2_Fc-IL-37 groups had less severe liver damage than the rIL-37 group due to the administration of a small dose. Similarly, liver egg granuloma was significantly inhibited in the CPP-IgG_2_Fc-IL-37 and no CPP-IgG_2_Fc-IL-37 groups compared with the rIL-37 group.

### IL-37 administration induces polarization of macrophage to M2 phenotype

**Fig. 3:**
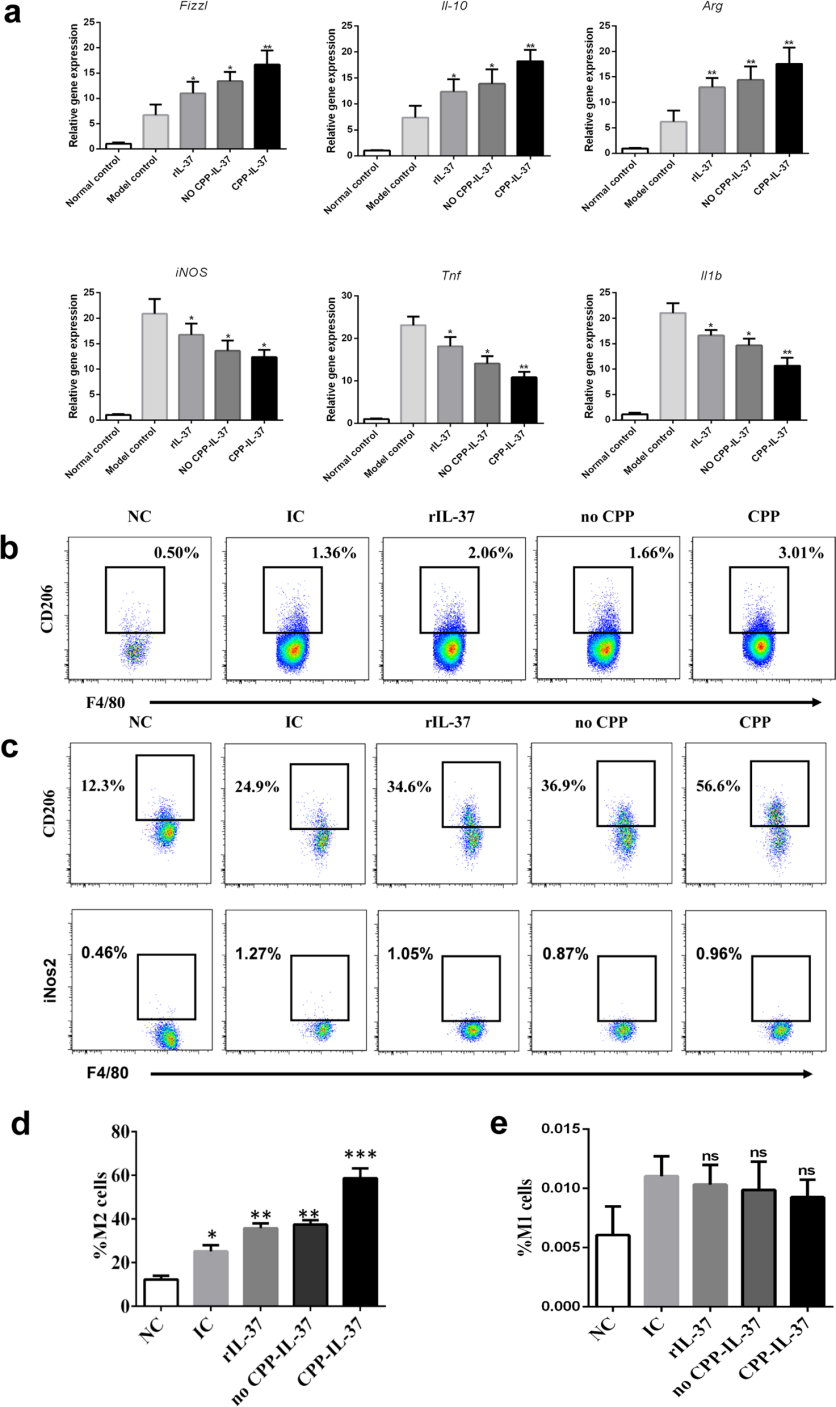
Administration of IL-37 induces polarization of macrophage to M2 phenotype. **a** Transcript levels for each chemokine tested (listed above each graph) expressed by macrophages were evaluated by quantitative real time-PCR. The messenger RNA levels were expressed relative to the levels in infected model controls following normalization with β-actin. All data are expressed as the mean ± SD, 5 mice per group. Asterisks indicate significant difference at *P < 0.05, **P < 0.01, versus infected model control. **b** FACS analysis of the ratio of M2 macrophages in the liver egg granuloma of the mice. **c** FACS analysis of M1 and M2 type macrophages in the liver egg granuloma of the mice. **d** Statistical analysis of M2 and M1 ratio by FACS analysis. Data represent the mean ± SD from three independent experiments, 5 mice per group. Asterisks indicate significant difference at *P < 0.05, **P < 0.01, ***P < 0.001. Abbreviations: FACS, Fluorescence-activated cell sorting; M1, M2, M1 and M2 macrophages, respectively; NC, Normal mice group; IC, infection model control group

The administration of IL-37 markedly attenuated the expression of the chemokines *iNOS* (t-test, *t*_(8)_ = 2.48, *P* = 0.038), *Tnf* (t-test, *t*_(8)_ = 4.50, *P* = 0.0020) and *Il1b* (t-test, *t*_(8)_ = 4.623, *P* = 0.002) in M1 macrophages, while M2 macrophage-secreted chemokines, such as *Fizzl* (t-test, *t*_(8)_ = 2.57, *P* = 0.033), *Arg* (t-test, *t*_(8)_ = 3.353, *P* = 0.033) and *Il10* (t-test, *t*_(8)_ = 6.521, *P* = 0.000), were significantly increased in the group administered IL-37 (**P < 0.01, *P < 0.05; Fig. 3a). Fluorescence-activated cell sorting (FACS) was then used to analyze the ratio of macrophages in liver granulomas of different groups. The results showed that the number and ratio of M2 macrophages in liver granulomas were significantly higher in the CPP-IgG_2_Fc-IL-37 group than in the other two IL-37 groups and the infection groups (Fig. 3b). The ratio of M1 and M2 macrophages in liver granulomas was also analyzed using FACS. When infected mice were administered rIL-37, CPP-IgG_2_Fc-IL-37 or no CPP-IL-37, the ratio of M2 increased more than in the infection groups. Among these, the proportion of M2 increased the most in the CPP-IgG_2_Fc-IL-37 group (by 56.6%; ***P < 0.001, **P < 0.01, *P < 0.05; Fig. 3c, d). However, the ratio of M1 macrophages in these groups did not change significantly (Fig. 3c, e).

Taken together, these results may suggest that IL-37 administration could induce polarization of macrophages to the M2 phenotype and suppress inflammation.

### IL-37 promotes the polarization of peritoneal macrophages to M2 phenotype in vitro

**Fig. 4:**
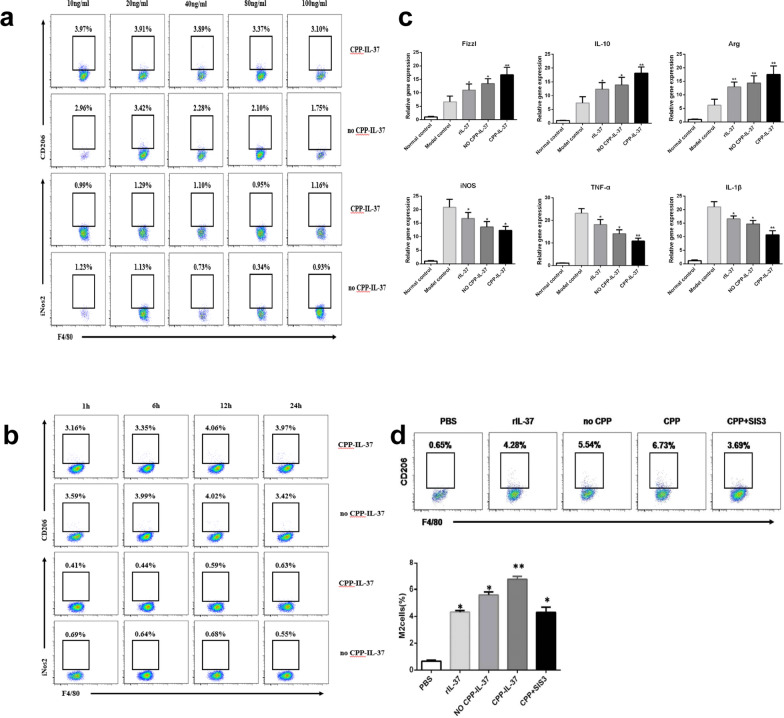
IL-37 promotes the polarization of peritoneal macrophages to the M2 phenotype in vitro. Peritoneal macrophages were purified from mice infected with *S. japonicum* or from the IL-37 treatment group. **a** Optimum protein content of CPP-IL-37 protein and no CPP-IL-37 protein in vitro was determined using FACS. The stimulation time was 24 h. The concentrations of CPP-IL-37 protein and no CPP-IL-37 protein were 10, 20, 40, 80 and 100 ng/ml, respectively. Data represent the mean ± SD from three independent experiments, with 5 mice per group in each experiment. **b** Determination of the optimum time of CPP-IL-37 protein and no CPP-IL-37 protein in vitro by FACS. The concentrations of CPP-IL-37 protein and no CPP-IL-37 protein were 10 and 20 ng/ml, respectively. The stimulation time was 1, 6, 12 and 24 h, respectively. Data represent the mean ± SD from three independent experiments, with 5 mice per group in each experiment. **c** Transcript levels for each chemokine in peritoneal macrophages were evaluated by qRT-PCR. The mRNA levels were expressed relative to the levels in the IL-37 protein treatment group following normalization with β-actin. All data are expressed as mean ± SD. Asterisks indicate significance at *P < 0.05, **P < 0.01. **d** The ratio of peritoneal M2 macrophages polarized in vitro. The cells were co-cultured with rIL-37, no CPP-IL-37, CPP-IL-37 + SIS3 or CPP-IL-37 protein for 12 h, respectively. Cells were labeled with F4/80 and CD206. Data represent the mean ± SD from three independent experiments, with 5 mice per group in each experiment. Abbreviations: PBS, phosphate-buffered saline (control); SIS3, SIS3, selective Smad3 inhibitor

The mechanism underlying how IL-37 induces the phenotypic shift in macrophages to the M2 phenotype is currently unclear and requires further study. To elucidate this, we first isolated and cultured peritoneal macrophages of C57BL/6 mice. The optimal concentration and time required by CPP-IgG_2_Fc-IL-37 and no CPP-IgG_2_Fc-IL-37 proteins to promote the polarization of mice peritoneal macrophages to the M2 phenotype were measured in in vitro experiments (Fig. 4a, b). Following the stimulation of macrophages with 10 ng/ml CPP-IgG_2_Fc-IL-37 protein or 20 ng/ml no CPP-IgG_2_Fc-IL-37 protein for 12 h, the greatest ratio of M0 to M2 polarization was 4.1% and 4.0%, respectively, which resulted in attenuation of the expression of the chemokines *iNOS* (t-test, *t*_(8)_ = 2.54, *P* = 0.035), *Il1b* (t-test, *t*_(8)_ = 13.29, *P* < 0.0001) and *Tnf* (*t-test, t*_(8)_ = 16.44, *P* < 0.0001) in M1 macrophages when peritoneal macrophages were treated with different doses of IL-37 proteins. However, the expressions of the chemokines *Fizzl* (t-test, *t*_(8)_ = 12.49, *P* < 0.0001), *Arg* (t-test, *t*_(8)_ = 17.42, *P* < 0.0001) and *Il10* (t-test, *t*_(8)_ = 11.58, *P* < 0.0001), secreted by M2 macrophages, were significantly increased (**P < 0.01, *P < 0.05; Fig. 4c). At this time, there was no obvious change in the proportion of M1 macrophages. Subsequently, the macrophages were co-cultured with 100 ng/ml rIL-37, 20 ng/ml no CPP-IgG_2_Fc-IL-37, 10 ng/ml CPP-IgG_2_Fc-IL-37 or 10 ng/ml CPP-IgG_2_Fc-IL-37 with 2 μM SIS3 or PBS for 12 h. The ratio of M2 macrophages was analyzed by FACS, with the results showing that the proportion of M0 macrophages polarized to M2 macrophages was significantly increased compared with the PBS group (**P < 0.01, *P < 0.05; Fig. 4d). There was no statistical difference in the ratio of M2 macrophages among the rIL-37, no CPP-IgG_2_Fc-IL-37 and CPP-IgG_2_Fc-IL-37 with SIS3 groups. The ratio of M2 macrophages was 6.73% in the CPP-IgG_2_Fc-IL-37 group, but this ratio was found to be increased in the other groups (**P < 0.01, *P < 0.05; Fig. 4d). These results indicate that CPP-IgG_2_Fc-IL-37 protein can induce the phenotypic polarization of macrophages to M2 macrophages through cell surface receptor signaling pathways and nuclear transcription factor pathways.

### IL-37 induces M2 polarization by promoting the expression of phosphorylated AMPK in macrophages

**Fig. 5:**
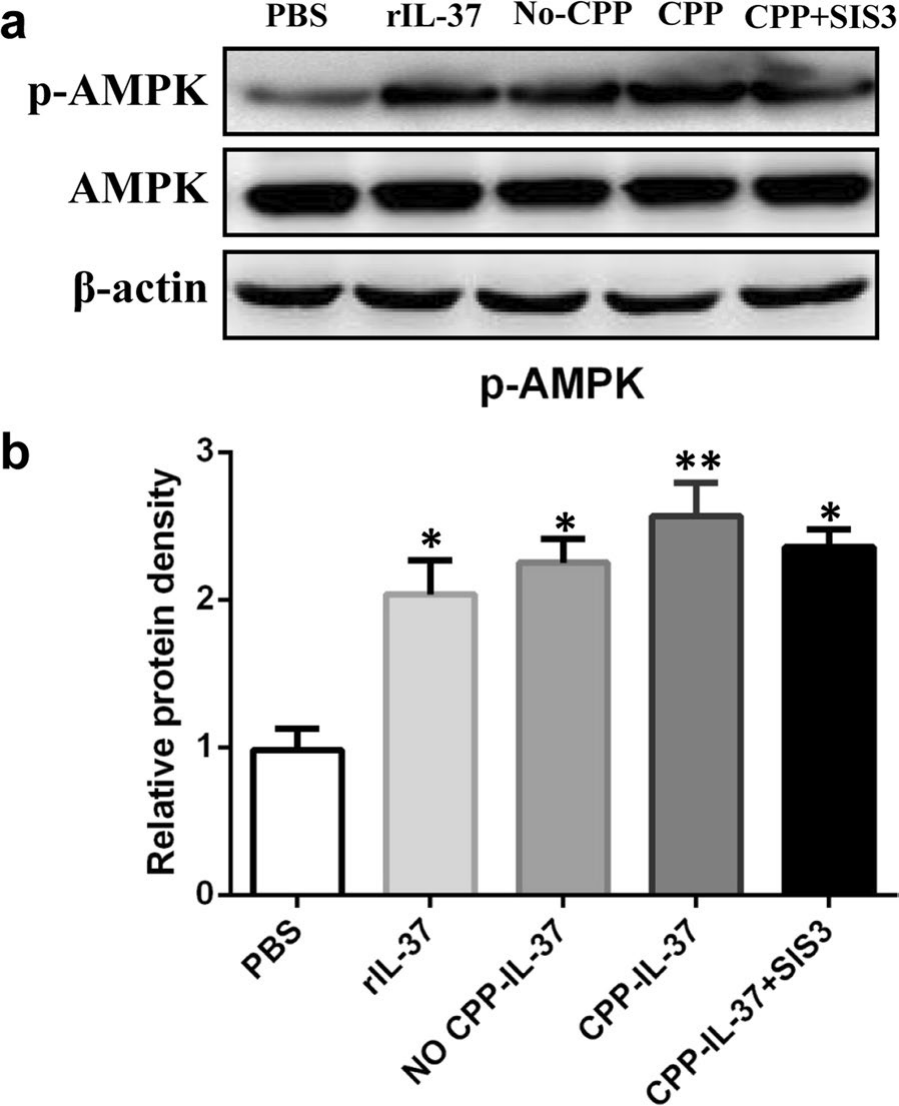
IL-37 induces M2 polarization by promoting the expression of pAMPK in macrophages. **a**, **b** Peritoneal macrophages were purified from mice infected with *S. japonicum* or from the IL-37 treatment group. The peritoneal macrophages were co-cultured with rIL-37, no CPP-IL-37, CPP-IL-37 + SIS3 or CPP-IL-37 protein for 12 h, respectively. Western blotting was used to analyze the expression of pAMPK and AMPK in different groups. Data represent the mean ± SD from 3 independent experiments. Asterisks indicate a significant difference at *P < 0.05, **P < 0.01

As described earlier, macrophages were co-cultured with 100 ng/ml rIL-37, 20 ng/ml no CPP-IgG_2_Fc-IL-37, 10 ng/ml CPP-IgG_2_Fc-IL-37, 10 ng/ml CPP-IgG_2_Fc-IL-37 and 2 μM SIS3, or PBS for 12 h. Western blotting was using to detect the expression of phosphorylated AMPK (pAMPK) and AMPK in each group. The expression of pAMPK was increased in the IL-37 protein treatment groups compared with the PBS group. Among the IL-37 protein treatment groups, the expression of pAMPK in the CPP-IgG_2_Fc-IL-37 group increased the most. However, the expression of pAMPK after SIS3 inhibition in the CPP-IgG_2_Fc-IL-37 group was similar to that in the rIL-37 and no CPP-IgG_2_Fc-IL-37 groups. Subsequently, an AMPK inhibitor, Compound C, was used to suppress AMPK activation. We observed the polarization of macrophages to determine whether IL-37 induces M2 macrophage polarization by promoting the expression of pAMPK in macrophages. We found that when macrophages were induced by CPP-IgG_2_Fc-IL-37 protein for 12 h, the ratio of macrophages polarized to M2 phenotype was significantly increased compared with the dimethyl sulfoxide (DMSO) group. Once macrophages had been pre-treated with different concentrations of Compound C, the ratio gradually decreased with increases in Compound C concentration (**P < 0.01, *P < 0.05; Fig. 5). The result demonstrated that M0 macrophages were no longer polarized to M2 macrophages following the inhibition of AMPK activation, indicating that IL-37 promotes the polarization of macrophages to the M2 phenotype by activating AMPK.

## Discussion

IL-37, a special member of IL-1 family, can generally suppress innate immunity and acquired immunity and, consequently, it plays an important role in a variety of inflammatory and autoimmune diseases [18]. However, the specific mechanism of IL-37 still needs to be explored in depth; in particular, its role in schistosome infection has not yet been reported. The aim of the present study was, therefore, to observe the role and mechanism of IL-37in host liver egg granuloma and hepatic fibrosis.

In inflammatory diseases, increased levels of endogenous IL-37 protein have a protective effect on the body, with the ability to suppress inflammation in gout, atherosclerosis and chronic periodontitis [19–21]. Our study found that the level of IL-37 in the serum of patients with acute schistosomiasis infection was significantly increased, suggesting that IL-37 expression is an anti-inflammatory response through a certain negative feedback regulation mechanism. It has a specific relationship with the occurrence and progression of schistosomiasis.

Unlike other members of the IL-1 family, the gene encoding IL-37 in mice has not yet been identified, making it impossible to study its endogenous function in mice. Therefore, to reveal the anti-inflammatory properties of IL-37, we constructed mice disease models using human IL-37 transgenic mouse models, such as endotoxemia, colitis and spinal cord injury and metabolic syndrome mice [8, 11, 18, 22]. Similarly, IL-37 also has a protective effect in mouse models of endotoxemia, acute lung injury, and asthma when treated with rIL-37 protein [11, 23, 24]. Since the currently commercially available rIL-37 cytokine is a prokaryotic expression protein, it lacks proper post-translational modifications and does not reflect the role of its transcription factors, which may have led to an underestimation of its draggability. A second aim of our study was to determine how IL-37 inhibits the formation of host liver egg granuloma and hepatic fibrosis and its mechanism. To this end, the recombinant CPP-IgG_2_Fc-IL-37 and no CPP-IgG_2_Fc-IL-37 eukaryotic expression proteins were independently designed and prepared by Professor Wenda Gao, one of the main members of our team. CPP-IgG_2_Fc-IL-37 can deliver both cell surface signals and nuclear factor signals, while no CPP-IL-37 only binds to the IL-37 receptor on the cell surface to activate the receptor signaling pathway because it has no CPP and protease cleavage site. The mechanism of IL-37 through cell surface membrane receptors, nuclear transcription factor pathways or both could be fully studied due to the novel design of this experiment that combines the IL-37 protein with CPP and IgG fusion protein.

The CPP-IgG_2_Fc-IL-37, no CPP-IgG_2_Fc-IL-37 and rIL-37 proteins were used simultaneously in our study. The results of the pharmacokinetics experiments suggest that the concentration of IL-37 in the liver was similar to that after administration of 5 ng/kg CPP-IgG_2_Fc-IL-37, 10 ng/kg no CPP-IgG_2_Fc-IL-37 and 20 ng/kg rIL-37 proteins. All three recombinant IL-37 proteins were able to suppress the formation and development of host liver egg granulomas and hepatic fibrosis following administration of the the above-mentioned doses. Among these, the effect of CPP-IgG_2_Fc-IL-37 protein was better than that of the no CPP-IgG_2_Fc-IL-37 and rIL-37 proteins. Since the doses of the CPP-IgG_2_Fc-IL-37 and no CPP-IgG_2_Fc-IL-37 proteins were lower than that of the rIL-37 protein, the corresponding liver damage was reduced. Our findings indicate that the CPP-IgG_2_Fc-IL-37 and no CPP-IgG_2_Fc-IL-37 proteins have good therapeutic effect and fewer side effects.

It has been reported that IL-37 can switch the polarization of macrophages from a pro-inflammatory M1 phenotype to a beneficial anti-inflammatory M2 phenotype in temporomandibular joint inflammation [15], but this mechanism of IL-37 in schistosomiasis has not yet been studied. Following rIL-37 protein administration in *S. japonicum*-infected mice, the expression of *iNOS* in the liver tissue was significantly downregulated, whereas that of *Fizzl* was upregulated. Similarly, the number and ratio of M2 macrophages in mice liver egg granuloma cells and peritoneal macrophages were also significantly increased. These results reveal that IL-37 may suppress liver egg granuloma and hepatic fibrosis by inducing macrophages to polarize to the M2 phenotype.

AMPK is a highly conserved intracellular energy sensor that plays a key role in regulating glucose, lipid and protein metabolism [25, 26]. In addition, AMPK is known to exert a central anti-inflammatory effect in inflammatory rheumatoid arthritis, non-alcoholic steatohepatitis and other inflammatory and autoimmune diseases [27, 28]. The expression level of mTOR, a serine/threonine protein kinase, has been known to decrease, whereas that of AMPK to increase in bone marrow macrophages isolated from IL-37-tg mice [11]. Furthermore, activated AMPK could help regulate the inflammatory response by promoting the polarization of macrophages to the M2 phenotype [29]. Our results show that the three recombinant IL-37 proteins were able to promote the activation of AMPK in mice peritoneal macrophages. All three recombinant proteins also promoted the polarization of macrophages derived from liver egg granuloma and abdominal cavity in *S. japonicum*-infected mice to the M2 phenotype, with the CPP-IgG_2_Fc-IL-37 protein exhibiting the strongest effect. Thus, our results indicate that IL-37 induces the polarization of macrophages to the M2 phenotype via promotion of AMPK activation, ultimately reducing the development of liver egg granuloma and hepatic fibrosis.

Mature IL-37 can not only be secreted as an extracellular cytokine but can also translocate into the nucleus to affect gene expression [18, 23, 30]. Luo et al. found that IL-37b is able bind to SMAD3 and inhibit the SMAD pathway by interfering with the formation of the SMAD2/3/4 complex and nuclear translocation in tumor cells [31]. Its biological effect depends on the activation of SMAD3 when IL-37 acts as a nuclear factor. Moreover, the IL-37/SMAD3 pathway can inhibit the production of inflammatory factors [11]. The CPP-IgG_2_Fc-IL-37 protein used in our study was able to deliver both cell surface and intracellular and nuclear factor signals;s the no CPP-IgG_2_Fc-IL-37 protein was only able to bind to the cell surface IL-37 receptor; and the rIL-37 The protein was not able to penetrate the cell membrane. The results of FACS and qRT-PCR showed that the CPP-IgG_2_Fc-IL-37 group had the largest proportion of cells induced by M2 macrophages. Western blotting demonstrated that activation of AMPK was more significant in the CPP-IgG_2_Fc-IL-37 group than in the other groups. These results suggest that the CPP-IgG_2_Fc-IL-37 protein could induce the polarization of macrophages to the M2 phenotype not only through the cell surface receptor signaling pathway but also through the nuclear transcription factor pathway. This study used the CPP-IgG_2_Fc-IL-37 protein with independent intellectual property rights for the first time, confirming that a lower working concentration of CPP-IgG_2_Fc-IL-37 can exert a better effect than the currently marketed rIL-37.

## Conclusions

The results of our study suggest that IL-37-induced AMPK activation through its interaction with SMAD3 results in promotion of the polarization of macrophages to the M2 phenotype and ultimately to the reduction of liver egg granuloma and hepatic fibrosis formation in schistosomiasis. Moreover, the CPP-IgG_2_Fc-IL-37 protein with independent intellectual property rights was found to be effective at smaller doses and exhibited fewer side effects and a better curative effect than the commercially available rIL-37 protein. These findings provide a foundation for the development of IL-37-Ig fusion protein as a novel treatment for various immune diseases in the future.

## Supplementary Information

**Additional file 1: Table S1.** Parasite burden, egg burden and liver weight of different groups of mice.

## Data Availability

All data generated during this study are included in this published article and its additional information files.

## Abbreviations

ALT: Alanine aminotransferase
AST: Aspartate aminotransferase
AMPK: AMP-activated protein kinase
CPP: Cell-penetrating peptide
IL-37: Interleukin-37
iNOS: Inducible nitric oxide synthase
