# Supplementary material for: IL-37 alleviates liver granuloma caused by Schistosoma japonicum infection by inducing alternative macrophage activation
Source: Parasit Vectors. 2022 Aug 24;15:300. doi: 10.1186/s13071-022-05420-6 (PMC9404629; doi:10.1186/s13071-022-05420-6)
Supplement: Supplementary file 1 — Additional file 1: Table S1. Parasite burden, egg burden and liver weight of different groups of mice. [file 13071_2022_5420_MOESM1_ESM.docx]

**Additional file 1: Table S1.** Parasite burden, egg burden and liver weight of different groups of mice (*n*=10)

|  | Normal control | Infection model control | | rIL-37 | No-CPP-IL-37 | | CPP-IL-37 |
| --- | --- | --- | --- | --- | --- | --- | --- |
| Worms/mouse | 0 | | 17±3.61 | 16±3.89 | | 17±3.94 | 17±3.52 |
| Egg/liver (×10^4^) | 0 | | 10±7.51 | 11±6.17 | | 10±7.56 | 11±6.49 |
| Liver weight | 1.99±2.22 | | 1.79±2.22 | 1.63±2.25 | | 1.62±2.47 | 1.61±2.23 |
